# A case of me: clinical cancer sequencing and the future of precision medicine

**DOI:** 10.1101/mcs.a000349

**Published:** 2015-10

**Authors:** Lukas D. Wartman

**Affiliations:** Washington University School of Medicine, St. Louis, Missouri 63110, USA

## Abstract

In this invited Perspective, I detail how my own experience as a patient with acute lymphoblastic leukemia (ALL) exemplifies several key concepts central to the implementation of cancer sequencing and precision medicine into clinical practice.

The field of precision medicine continues to rapidly grow and evolve as a result of the tremendous advances in sequencing technology, the ongoing improvement in genomic data analysis, and the decreasing cost associated with both sequencing data generation and analysis. The overarching principle that defines precision medicine is for health-care providers to optimize the treatment of patients, regardless of their disease, based on the results of comprehensive testing that can reliably and robustly assay for the relevant markers of individual variability that are pertinent for successfully eradicating a given disease, while minimizing the toxicity of the therapy to the patient. Certainly, clinical genomics, irrespective of the sequencing platform, is not synonymous with precision medicine as many other factors weigh into applying individualized therapy to a particular patient, such as environmental exposures and the patient's own treatment preferences. Nonetheless, the potential for clinical sequencing results to guide treatment choices is clear. Moreover, in the future, the results of sequencing studies may provide the basis for targeted efforts for disease prevention in patients found to harbor specific susceptibility alleles.

My own experience as a patient with acute lymphoblastic leukemia (ALL) exemplifies several key concepts central to the implementation of cancer sequencing and precision medicine into clinical practice. I was diagnosed with ALL now over a decade ago, in 2003, while I was finishing my medical school training at Washington University School of Medicine in St. Louis. I was treated “off-study” because even our large cancer center did not have an open clinical trial for adult patients with a new diagnosis of ALL at the time. It remains true that the majority of adult patients with ALL (and other cancers) are treated off-study in the United States, whereas nearly all pediatric patients with ALL (and other cancers) are treated on an active clinical trial protocol (Gelijns and Gabriel 2012). I was treated with a multidrug chemotherapy regimen that was based on a trial that started to accrue patients in 1988, nearly two decades before my own diagnosis (Larson et al. 1995). However, in comparison to the treatment of most other cancers, the therapy for ALL, although dated, was far from imprecise. Instead, many of the drugs used to treat the disease target ALL lymphoblasts, more or less specifically, and are not active in the treatment of other cancers, including acute myeloid leukemia (AML). These drugs include the antimetabolites methotrexate, 6-mercaptopurine, and thioguanine; steroids; the vinca alkaloid vincristine, which interferes with the assembly of microtubules; and the enzyme l-asparginase, which depletes asparagine, an essential amino acid for ALL lymphoblasts but not for normal noncancerous cells.

Pediatric oncologists used combinations of these drugs together with conventional chemotherapy in well-designed clinical trials to achieve cure rates that now approach 90% in children with the disease (Hunger et al. 2012). Unfortunately, the cure rate for adults, even with modern regimens that approximate these treatment regimens, still significantly lags behind that of children. Current pediatric-inspired regimens cure ∼50% of adults younger than age ∼40, whereas older patients have much poorer outcomes (Rowe et al. 2005; Huguet et al. 2009; Thomas et al. 2010). Several factors have traditionally been associated with the difference between the outcomes seen in pediatric versus adult ALL patients. These factors include the increasing incidence with age of Philadelphia Chromosome t(9;22)–positive disease, which was associated with a poor prognosis until tyrosine kinase inhibitors that target BCR-ABL were incorporated into treatment regimens for this patient population (Thomas et al. 2004; Yanada et al. 2006; Ravandi et al. 2010; Foa et al. 2011; Mizuta et al. 2011); the inability of adult patients to tolerate equivalent doses of chemotherapy in pediatric regimens without undue toxicity; and the trend for adults to be treated off-study without adherence to the strict protocol requirements that are built into pediatric trials. However, even when these poor prognostic factors are considered, adult patients still fare worse than would be expected. One plausible explanation is that there remain uncharacterized differences, other than the incidence of t(9;22), in the genetics of adult ALL that render the disease resistant to current treatment regimens.

At the time of my diagnosis, my oncologist ordered routine cytogenetics and a panel of FISH testing from my initial bone marrow biopsy. Even today, no further molecular diagnostic testing has been demonstrated to have prognostic or therapeutic significance for adult patients with ALL. Both the cytogenetics and FISH from my leukemia sample showed a deletion of Chromosome 12p, which has no known prognostic or therapeutic significance. Minimal residual disease (MRD) testing was not widely used at that time so no MRD assessment was performed during my initial course of therapy. A prospective study had been published linking MRD and the risk of relapse in adult patients with ALL so it would have been reasonable then to modify my management if I were MRD-positive at the end of consolidation and move forward with a matched sibling allogeneic transplant rather than receive standard maintenance therapy (Mortuza et al. 2002). However, given that I did not have any known unfavorable risk factors and was relatively young (<30 yr old) at the time of my diagnosis, the chances that I would be cured without the additional risk of an allogeneic transplant were better than the average adult (and even young adult).

Nonetheless, 5 years after my initial diagnosis, in 2008, I relapsed. I was treated with aggressive chemotherapy, entered again into a complete morphologic remission (FISH was also negative for the 12p deletion, which was present at diagnosis and at relapse), and had a bone marrow transplant with a myeloablative conditioning regimen (Marks et al. 2006). My younger brother, who is an HLA-matched sibling, was the donor. Although the transplant was not without significant toxicity, over the course of the year after the transplant, my health returned to near baseline, and I had no significant complications from the treatment. I returned to work and completed my clinical rotations as an oncology fellow.

“…my case is not worth sharing because of my good luck, but rather because it illustrates how cancer genomics and precision medicine can be clinically relevant.”

**Figure:**
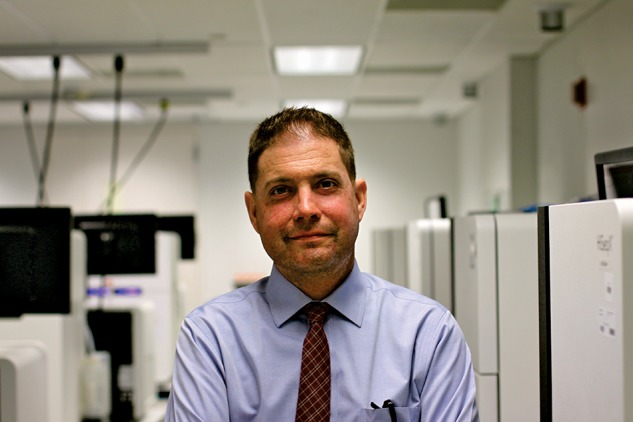
Dr. Lukas Wartman photographed next to an Illumina HiSeq X Ten sequencer at The Genome Institute at Washington University School of Medicine. Photo by Josh Peck.

I then joined Dr Timothy Ley's laboratory to begin my postdoctoral work as a research fellow. I had been fascinated by genetics and cancer since my undergraduate studies so joining Tim's laboratory was a natural fit and not directly related to my own cancer diagnosis. At the time, Tim and colleagues at The Genome Institute at Washington University School of Medicine were finishing work on sequencing the first cancer genome from a patient with AML (Ley et al. 2008). While I was involved peripherally with the human AML sequencing project, my own work focused on sequencing tumors derived from a mouse model of AML in an attempt to better understand the progression events that lead to AML when the initiating event is known (Wartman et al. 2011). All of the sequencing work done at that time was grounded by our group's rationale that to understand the pathogenesis of AML, researchers had to define the specific genetic events that drive the disease (both somatic and germline variants). Sequencing AML genomes was a relatively comprehensive and unbiased approach used to identify candidate variants responsible for disease pathogenesis. Yet from the beginning of this endeavor, it was clear that some somatic variants were initiating events and others were progression events. To design an effective targeted therapy to eradicate AML, we reasoned that a drug would have to target the initiating event(s) present in the “founding” clone and not merely target progression events present in subclones (Ding et al. 2012). Moreover, the relevance of many variants to AML pathogenesis was and, to a certain extent, remains unclear. In short, the initial AML sequencing studies aimed to define the genetic “landscape” of the disease, which included identifying the significantly recurrently mutated genes and the relationships among different genetic mutations in a given case (Mardis et al. 2009; Ley et al. 2010; Welch et al. 2012; Cancer Genome Atlas Research Network 2013). This work was “discovery” genomics, intended to generate hypotheses that could be experimentally validated through a variety of approaches in the wet laboratory. Once validated, a particular alteration could then be targeted therapeutically. As a cancer survivor and physician-scientist, I saw (and continue to see) great promise in this step-wise approach from discovery work to experimental validation to translational targets that spur drug development.

Things changed when I relapsed again in 2011. I was treated with high-dose conventional chemotherapy on a clinical trial that incorporated an agent intended to disrupt the bone marrow stromal environment and render the leukemic cells more sensitive to chemotherapy (G Uy and D Link, unpubl.). Unfortunately, I experienced significant toxicity from the regimen and did not enter into a remission. I had exhausted all of the standard treatment options for my relapsed, refractory disease and could not tolerate further aggressive chemotherapy—nor were there promising clinical trials open anywhere in the world at the time. The overall survival of adults with ALL after first relapse is ∼5%–10%, as reported by several large retrospective studies (Fielding et al. 2007; Oriol et al. 2010). Most oncologists consider adult ALL in second relapse to be an incurable, and unvaryingly fatal, disease. And this appeared to be my fate as well.

However, at the time of my second relapse, a research protocol had recently opened at Washington University School of Medicine for the genomic sequencing of patients with lymphoid malignancies, including ALL. Tim offered me a spot on the protocol, and I consented to have a leukemic sample banked from a bone marrow biopsy before the administration of any chemotherapy. Drs Tim Ley, Elaine Mardis, and Richard Wilson decided to move forward with whole-genome sequencing (WGS), concurrent exome sequencing, and RNA-seq of my relapsed leukemia sample (as well as a skin sample to use as germline comparator for the DNA sequencing studies). I elected to participate because I believed that sequencing my leukemia genome, the first adult ALL sample to be sequenced, could be the first step for researchers to understand why adults with ALL fare worse than children. My primary oncologist, Dr John F. DiPersio, supported the endeavor with similar reasoning; although, he also recognized that the sequencing data could reveal a cryptic t(9;22) translocation that was not detected by conventional cytogenetics or FISH but would be clinically actionable if present. In my mind, however, the sequencing of my leukemia genome was “discovery” genomics. I did not expect the results to be clinically important for me.

“…my oncologist called me about a week later to tell me that Malachi had identified a clinically actionable target from the RNA-seq data…”

Yet, after the chemotherapy failed to achieve a remission, I quickly realized that identifying a novel target from the sequencing results was likely my only chance at survival. My primary oncologist relayed the results of the WGS and exome sequencing studies to me just after I found out that I had persistent leukemia after my course of failed induction chemotherapy and ∼4 wk after I was diagnosed with relapsed disease. A team of genomic analysts led by Dr Malachi Griffith (I was not involved in any way in the analysis or interpretation of my own sequencing data) identified approximately 50 nonsynonymous somatic mutations in my leukemia genome, but none of the mutations was “targetable” by FDA-approved drugs (or were even good targets of experimental drugs in clinical trials). The WGS data showed no evidence of a cryptic t(9;22). The RNA-seq data were still being processed, but no one held out much optimism that the results would be significantly different. It was a tremendous surprise when my oncologist called me about a week later to tell me that Malachi had identified a clinically actionable target from the RNA-seq data: My leukemia cells were massively overexpressing wild-type *FLT3*, a receptor tyrosine kinase important for hematopoietic cell survival, development, and proliferation. Previously published work demonstrated that some ALL samples did have high *FLT3* expression and that ALL lymphoblasts in culture were sensitive to FLT3 inhibitors, but no clinical trial results of ALL patients had been reported using these drugs (Armstrong et al. 2002; Brown et al. 2005). And no clinical practitioners routinely screened for *FLT3* expression in patients with ALL. In short, the sequencing results uncovered a novel, unexpected target. However, it was still unclear whether my leukemia would respond to a FLT3 inhibitor—but there were no other reasonable options available to me at the time. I started taking the drug, sunitinib (Sutent, Pfizer), which is FDA-approved for other types of cancer, but is also known to be a potent wild-type FLT3 inhibitor. Remarkably, 2 wk later, I was in a complete remission and went on to have a second stem cell transplant from an unrelated matched donor—because it was unlikely that sunitinib alone would cure my disease. My posttransplant course has been complicated by significant graft-versus-host disease related to the transplant, and I was only able to take sunitinib for a short time after the transplant. However, I remain in complete remission more than 3 years later.

When I share my story, many people remark on how incredibly lucky I was to be working with Tim and affiliated with The Genome Institute, or how lucky I was that sunitinib actually worked to put my disease into remission, greatly increasing the likelihood of success of the second stem cell transplant. Both of these points are certainly valid; however, for me, my case is not worth sharing because of my good luck, but rather because it illustrates how cancer genomics and precision medicine can be clinically relevant. The important part of my own story is how incredible science, born out the Human Genome Project and the tremendous amount of pioneering work done since then at The Genome Institute and several other large sequencing centers, made the sequencing and analysis of my leukemia genome possible. Health-care providers now face the challenge of responsibly incorporating this new technology into broader clinical practice in an effort to help more patients and bring precision medicine into the forefront of health care (Feero et al. 2010). We are at the beginning of the learning curve on how we will move beyond *n* = 1 case reports to clinical trials that will incorporate clinical sequencing into the treatment of a wide variety of different disease types. Significant costs will be associated with this challenge: maintaining accredited sequencing laboratories and the costs of data interpretation will remain significant while the cost of sequencing itself has reached a plateau using current platforms. Today, as advanced molecular characterization of a wide spectrum of human disease is more broadly available, it is essential that researchers share insights garnered from case studies as the foundation for moving forward with the implementation of precision medicine as the standard of care—rather than the exception. Although the challenges remain substantial, the research and clinical communities must find the will and the resources to make this happen now. Not later. Now.

## Competing Interest Statement

The author has declared no competing interest.
